# Review of NVP and HG and Early Pharmacotherapeutic Intervention

**DOI:** 10.1155/2012/252676

**Published:** 2011-11-24

**Authors:** Shannon M. Clark, Maged M. Costantine, Gary D. V. Hankins

**Affiliations:** Division of MFM, Department of Obstetrics and Gynecology, University of Texas Medical Branch, 301 University Boulevard, Galveston, TX 77555, USA

## Abstract

NVP occurs in 50–90% of pregnancies, making it a common medical condition in pregnancy. Women present differently with any combination of signs and symptoms. It is appropriate to take the pregnancy-related versus nonpregnancy-related approach when determining the cause of nausea and vomiting but other causes should be considered. The most common etiologies for NVP include the hormonal changes associated with pregnancy, the physiologic changes in the gastrointestinal tract, and a genetic predisposition. Up to 10% of women will require pharmacotherapy to treat the symptoms of NVP despite conservative measures. ACOG currently recommends that a combination of oral pyridoxine hydrochloride and doxylamine succinate be used as first-line treatment for NVP if pyridoxine monotherapy does not relieve symptoms. A review of NVP and early pharmacotherapeutic management is presented due to the fact that NVP is largely undertreated, and investigations into the safe and effective pharmacotherapies available to treat NVP are lacking.

## 1. Introduction

Nausea and vomiting in pregnancy (NVP) is a very common and oftentimes difficult medical condition to manage in pregnancy. The spectrum of disease ranges from a limited, mild to moderate course that resolves with conservative treatment or with the addition of an antiemetic to a severe, prolonged course requiring multiple triage visits and/or hospital admissions and pharmacotherapy. Determining the appropriate intervention while keeping fetal exposure in mind can make management of the patient challenging. Furthermore, while early recognition and treatment of symptoms is ideal, communication between the pregnant woman and her heath care provider is often lacking, allowing for progression of symptoms. A review of NVP and early pharmacotherapeutic management is needed due to the fact that NVP is largely undertreated, and there is a lack of investigation into the safe and effective pharmacotherapies available to treat NVP. This allows pregnant women to be orphaned from the benefits of existing knowledge. As a result, review of NVP is presented here, along with recommendations for early pharmacotherapeutic management.

## 2. Definition and Incidence

NVP occurs in 50–90% of pregnancies, with nausea and vomiting in approximately 50–55% and nausea alone in 25% [1–7]. Although NVP has been commonly referred to as “morning sickness,” nausea can occur at any time of the day, last for varying periods of time, and occur with or without episodes of vomiting. The usual onset for NVP is between 4–9 weeks gestational age, with maximal symptoms at 12–15 weeks, and resolution by 20 weeks gestational age [1, 4, 8]. There are a small percentage of pregnant women (approximately 9–20%), however, who experience symptoms beyond 20 weeks gestational age and even throughout the remainder of the pregnancy [8, 9]. Although this particular group of women may indeed comprise a small percentage with prolonged symptomatology, they present a clinical dilemma with a great amount of time and effort utilized to rule out other potential causes of their nausea and vomiting. Overall, NVP typically follows a usual course with management consisting of conservative measures to hospitalization for more acute management.

HG occurs in 0.3–3% of pregnancies and is typically defined as severe and persistent nausea and vomiting with or without retching, with a loss of 5% or more of prepregnancy body weight, electrolyte abnormalities, ketonuria, dehydration, and potential vitamin or mineral deficiencies (i.e., thiamine) [1–7]. These patients often require multiple triage visits for intravenous fluid hydration and antiemetics, with inpatient admission in the more severe cases. A prolonged hospital stay with a trial of multiple pharmacotherapies, gastrointestinal rest, and hydration can be necessary. Vitamin supplementation of intravenous fluids is typically required, with the addition of thiamine to avoid the development of Wernicke's encephalopathy [10]. The decision to admit a patient for the treatment of NVP or HG is subjective. However, any woman who is ketotic and dehydrated should be hospitalized not only for treatment but to explore any other potential causes for nausea and vomiting. HG can precipitate from NVP that has been neglected or under-treated. As a result, early recognition and inquiry regarding symptoms by the health care provider is essential.

## 3. Diagnosis, Differential Diagnosis, and Maternal Morbidity

Although each woman with NVP can present differently, the symptoms predominantly include any combination of the following: nausea, gagging, retching, dry heaving, vomiting, and odor and/or food aversion [11–13]. Each woman usually has a certain precipitating factor that triggers the nausea and vomiting, that is, movement-induced, heartburn, food and/or odor triggers [14]. During initial history taking, questioning on the onset, timing, severity, and aggravating and alleviating factors may point to another cause for the nausea and vomiting. This information is also helpful when formulating a treatment plan. One of the most important aspects of the history is the duration of vomiting in order to assess the potential risk for Wernicke's encephalopathy due to thiamine deficiency.

If the diagnosis of HG is made, the patient should be evaluated for urinary ketones, BUN, creatinine, aspartate aminotransferase (AST) and alanine aminotransferase (ALT), amylase, and electrolytes [15]. Thyroid stimulating hormone (TSH) and free T4 (FT4) should also be checked as human chorionic gonadotropin (hCG) cross-reacts with thyrotropin and stimulates the thyroid gland [15]. As a result, thyrotropin is typically lower in these patients. In fact, the values of TSH and FT4 in patients with HG may be similar to that seen in Graves' disease, but without the clinical symptoms and findings of Graves' disease or thyroid antibodies [16]. This form of hyperthyroidism usually resolves without treatment by 20 weeks gestational age, and management of nausea and vomiting until then is as indicated.

The vomiting observed with NVP and HG can be quantified with a scoring system, the Pregnancy-Unique Quantification of Emesis scale (PUQE) [17]. Similar to the Rhodes Scale used for the assessment for nausea and vomiting in patients receiving chemotherapy, the PUQE scoring system was designed to focus on nausea and vomiting of pregnancy [17, 18]. The 12 hour PUQE scoring system assesses the severity of NVP by focusing on the number of hours of nausea and the number of episodes of retching and vomiting, as well as an overall well-being score in the 12 hours immediately before assessment [19]. The 24 hour PUQE scoring system, validated in 2009, was subsequently developed to account for global nausea and vomiting, including the time spent sleeping and the severity of symptoms throughout the first trimester [20, 21]. The PUQE scoring system has a minimum score of 3 and maximum score of 15 with a score of <6 suggesting mild HG, 7–12 moderate, and >13 severe NVP. This scoring system has been validated and shown to correlate with clinical outcomes such as rates of hospitalization and women's subjective feelings of well-being [19]. There is also a well-being score of 0 (the worst possible) to 10 (the best possible), which is a general self-perception score of physical and psychological health and a question on the amount of sleep including naps in a 24 hour period of time [17, 19]. The PUQE scoring system is helpful not only to qualify and quantify the nausea and vomiting, but to follow the response to treatment and improvement over time.

Regardless of when the patient presents, it cannot be assumed that nausea and vomiting is due to NVP. It is more appropriate to take the pregnancy-related versus nonpregnancy-related approach when determining the etiology. More often than not, if the patient initially presents before 10 weeks, it is likely NVP. However, a thorough history and physical exam is still required to elicit any potential contributing or confounding factors at any gestational age. If the diagnosis of NVP or HG is made, but there is poor response to initial interventions, an atypical presentation, or initial presentation after 9–10 weeks, other causes must be explored [3, 4, 6, 7].  Table 1 lists other potential causes of nausea and vomiting in pregnancy. If there is fever, a source of infection should be sought or if the history suggests a CNS abnormality, check for signs of raised intracranial pressure [11]. Specific signs such as peritoneal signs, RUQ pain, or jaundice should raise the suspicion for acute abdomen, preeclampsia, and acute fatty liver of pregnancy, respectively [11]. In addition, headache with nausea and vomiting can occur with dehydration, but preeclampsia should still be ruled out, especially if there is elevated blood pressure. Although epigastric pain and hematemesis is rare, if this is observed, a Mallory Weiss tear from prolonged vomiting or gastrointestinal ulcer may be the cause. Finally, heartburn and gastric reflux occurs in a significant number of pregnant women, and appropriate recognition and treatment of this particular condition may improve symptoms quite rapidly.

Prolonged nausea and vomiting in the setting of NVP or HG can lead to maternal vitamin deficiencies. As mentioned above, Wernicke's encephalopathy is a potential serious or fatal maternal complication and is due to severe vitamin B1 (thiamine) deficiency. Approximately 47% of patients with this condition will present with a history of prolonged nausea and vomiting along with the triad of abnormal ocular movements, ataxia, and confusion; an additional percentage will also have diplopia [22, 23]. Symptoms can also be more variable and include memory loss, apathy, decreased level of consciousness, or blurred vision [22]. Although this condition is reversible with prompt treatment, 60% of women will have residual impairment and there is a 37% fetal loss rate [22]. Because maternal serum thiamine levels are not useful in making the diagnosis, any pregnant woman who presents with prolonged nausea and vomiting and neurologic abnormalities should be empirically treated with intravenous thiamine. Deficiencies in vitamins B6 and B12 are rare and not as potentially serious, but can cause anemia and peripheral neuropathy associated with hematemesis, malnutrition, and psychological effects [23]. Vitamin K deficiency and coagulopathy can also occur, leading to an abnormal coagulation profile and bleeding [24].

One consequence of NVP and HG that is commonly neglected, especially when the focus is on treatment, is the psychosocial impact of this disorder. NVP can adversely affect family and social life, physical and mental health, employment, and can impose an economic hardship [25, 26]. This can range from missing days of work to termination of the pregnancy due to severe symptoms. Up to 25% of pregnant women have to change their normal daily activities due to symptoms [14]. In addition, these women have lower physical and social functioning and miss more days of work [27]. Furthermore, depression and anxiety can develop, which can make management of the patient more complicated. Koken et al. investigated the association between depression and anxiety in early pregnancy, and nausea and vomiting in a cross-sectional study of 230 women using the Hospital Anxiety and Depression Scale as a measure of anxiety and depression, and the Rhode's System for nausea and vomiting [28]. A significant correlation between Rhode's score and both anxiety and depression scores was found. Gestational age showed an inverse correlation with anxiety scores. They also found an association between anxiety and depression in early pregnancy and severity of NVP. They concluded that recognizing depression in the early stages of pregnancy might be a key step in assisting the mother. As a result, health care providers should assess the severity of NVP and address the psychological situations of the patient [28]. Depression and anxiety can contribute to and confound the symptoms of NVP and render treatment more challenging.

## 4. Etiology and Risk Factors

A commonly accepted etiology for NVP and HG is attributed to the hormonal changes that occur during pregnancy involving hCG, estrogen, progesterone, and thyroid hormones. These hormone levels change throughout pregnancy with the most marked changes occurring during the first trimester [29, 30]. Because hCG and TSH have a similar biomolecular structure, conditions that are associated with an increase in hCG or hyperthyroidism may result in more severe NVP or HG [29, 30]. Several studies have shown a significantly higher level of serum hCG in HG patients than in controls [29]. This is further supported by the peak incidence of HG occurring when the trophoblast is most actively producing hCG. As a result, in pregnancies with increased placental mass, that is, multiple gestations and molar pregnancy, there is an increased incidence of HG [31–34]. There is also a strong association with HG and abnormal thyroid function tests (TFTs) [23]. Physiologic stimulation of the thyroid gland is common in early pregnancy due to the structural similarity between hCG and TSH, as previously stated [23]. In fact, it is recommended that TFTs be obtained with the initial workup of nausea and vomiting. Although, the TFTs may be abnormal, treatment is not typically indicated as the TFTs will normalize as the pregnancy progresses. 

The physiologic changes in pregnancy notably involve the gastrointestinal (GI) tract. Not only is the GI tract anatomically affected by the enlarging uterus, but it is also affected by the hormonal influences during pregnancy. Changes within the GI tract include gastric dysrhythmia (tachygastria or bradygastria or both) or gastroparesis and abnormalities in gastric neural activity and smooth muscle function [35]. This is predominantly due to the influences of progesterone and estrogen on the GI tract. In addition, the enlarging uterus and displacement of the abdominal organs can lead to adjustment of the gastroesophageal junction and reflux or nausea and vomiting. These changes in the GI tract are more significant in women with preexisting GI disease including diabetic gastroparesis, gastroesophageal reflux disease (GERD), gastric bypass surgery, and inflammatory bowel disease, potentially resulting in more severe symptoms [35–37]. If the patient has a preexisting GI condition, the approach to management and potential pharmacotherapeutic treatment of NVP or HG must be tailored to the particular condition. 

Helicobacter pylori (H. Pylori) is a gram-negative flagellated spiral bacterium found in the stomach. It has long been established that prolonged infection with this organism causes chronic gastritis, duodenal and gastric ulceration, and gastric cancer. It is commonly treated with triple therapy consisting of two antibiotics and a proton pump inhibitor or H2 blocker [38]. More recently, H. pylori infection has been associated with more severe HG and NVP, with some studies showing a higher incidence of infection with H. pylori in women with HG than in normal pregnant controls [23]. In a systematic review and meta-analyses of case-control studies by Sandven et al. examining the association between H. pylori infection and HG, 25 case-control studies were identified [39]. They found that exposure to H. pylori is associated with an increased risk of HG. Another study by Guven et al. investigated the relationship between H. pylori infection and HG in early pregnancy through serologic and stool antigen tests in a prospective cross-sectional study on 40 women with HG and 40 controls at 7–12 weeks of pregnancy [40]. They found that the rate of serology-specific H. pylori IgG positivity was 80% in subjects with HG and 35% in controls—a significant difference. There was also a significant difference in the rate of H. pylori stool antigen test positivity, with a rate of 87.5% in subjects with HG and 62.5% in controls. They concluded that both serologic and stool antigen testing were good screening methods to identify those subjects in early pregnancy with H. pylori infection and HG [40]. If a patient presents with excessive symptoms of nausea and vomiting that persist beyond the second trimester, greater or longer than expected symptoms of nausea and vomiting and/or weight loss, testing for the presence of H. pylori may be indicated [38].

Much attention has been given to the role that genetics plays in the development and severity of NVP and HG. Not only are NVP and HG likely heritable diseases, but the severity of the disease appears to be associated with a genetic predisposition [41]. It appears that women are at the greatest risk if their mother or sister had NVP or HG, or if the patient herself had severe disease in a previous pregnancy [4, 33]. In a study by Fejzo et al. 2008, the prevalence of severe NVP and HG among relatives of affected individuals was explored [42]. 1224 self-reported cases of HG along with their family histories were used for the purpose of the study. Each subject completed an online survey administered by the Hyperemesis Education and Research Foundation between 2003 and 2006. Approximately 28% of cases reported that their mother had severe NVP or HG while pregnant with them and of the 721 sisters with pregnancy history, 137 (19%) had HG. In severe cases requiring total parenteral nutrition (TPN) or nasogastric tube (NGT) feeds, the proportion of affected sisters was 25% [42]. With these results, strong preliminary evidence for a genetic component to extreme NVP or HG was established. 

Several risk factors have been associated with NVP and HG, with the most recognized being multiple gestation, molar pregnancy, positive family history. It also appears that NVP and HG are more common in young, nulliparous, obese women. In those who have NVP later in pregnancy (after 20 weeks), the women are older, have higher parity and BMI, and are more likely to develop gestational diabetes [43]. Factors that worsen NVP include stress, lack of sleep, chronic H. pylori infection, peptic or duodenal ulcers, migraines [25, 44]. Prenatal vitamins are often cited by women as initiating exacerbating symptoms of NVP and HG [45]. A study by Louik et al. in 2006 investigated the potential risk factors for NVP occurrence, time of onset, and duration of disease [46]. They found that the overall risk of NVP was 67%, with the risk, timing of onset, and duration of NVP nearly identical for mothers of both normal and malformed infants. They also found that younger women, multiparas, and multiple gestations had increased risk for NVP. They did not find an association between NVP and increased prepregnancy weight, black race, or low education, or sex of the infant. Finally, a longer duration of NVP increased with increasing prepregnancy weight, and late-onset NVP subjects were more likely to be less educated and have lower incomes [46].

## 5. Early Pharmacotherapeutic Intervention

It is estimated that up to 10% of women will require pharmacotherapy to treat the symptoms of nausea and vomiting despite changes in lifestyle and nutrition [15, 47]. The American College of Obstetricians and Gynecologists (ACOG) currently recommends that a combination of oral pyridoxine hydrochloride (vitamin B6) and doxylamine succinate be used as first-line treatment for NVP if pyridoxine monotherapy does not relieve symptoms (Table 2) [4]. Pyridoxine is a water soluble vitamin that is involved in the metabolism of amino acids, lipids, and carbohydrates [48]. Doxylamine is a histamine-1 (H1) receptor antagonist marketed in the USA as Unisom Night Time Sleep Aid (25 mg) that is typically given with pyridoxine. Doxylamine directly inhibits the action of histamine at the H1-receptor, acts indirectly at the vestibular system, and exhibits some inhibition of muscarinic receptors to decrease stimulation of the vomiting center [49, 50]. Although these medications are available individually over-the-counter in the United States (USA), in Canada the combination is available as Diclectin (Duchesnay, Inc., Lava QC, Canada), a sustained-release formulation of 10 mg of pyridoxine and 10 mg doxylamine [50]. It is currently recommended that 4 tablets of Diclectin be given a day. This dosage can be adjusted for severity of symptoms and/or maternal BMI. If the patient has a higher BMI, she may take up to 8–12 tablets a day without increasing maternal adverse effects, fetal risk, degree of tiredness, and birth defects [51]. These higher doses appear to be more efficacious. As symptoms improve and then resolve, patients should be tapered off Diclectin to avoid recurrence of symptoms [13].

The combination of pyridoxine and doxylamine has the most data on safety and efficacy, including data in the first trimester [4, 7, 52, 53]. It was originally available in both the USA and Canada as Bendectin, a delayed-release combination of doxylamine and pyridoxine. Bendectin had a similar formulation to Diclectin in that they contained the same active ingredients, but Diclectin utilizes modern manufacturing technology for a delayed-release tablet [54]. Bendectin was removed from the US market in 1983 due to allegations of teratogenicity, including fetal heart and limb reduction defects [1, 13, 55]. This left no available FDA-approved medication for the treatment of NVP in the USA Since its removal from the US market, the incidence of hospitalization for HG has increased 2-3-fold while the incidence remains unchanged in areas where Diclectin is now available, that is, namely Canada and Europe, and the rate of birth defects in the USA has not changed since its withdrawal [1, 4, 56, 57]. Despite the controversy and the fact that it is no longer available, Bendectin is still the most studied drug in pregnancy and no evidence shows a relationship between Bendectin use in the first trimester and congenital anomalies [58]. In Canada, Diclectin has been associated with a decreased incidence of hospitalization for NVP in observational studies [57, 59]. Furthermore, the combination of over-the-counter oral vitamin B6 and Unisom in the USA has been studied in over 6000 patients and controls with no evidence of teratogenicity, and in randomized trials it has been associated with a 70% reduction in nausea and vomiting [4, 60]. Many case-control and cohort studies, including over 170000 exposures, have demonstrated the safety of doxylamine and pyridoxine [48]. 

Despite the fact that allegations of teratogenicity have been unsubstantiated, no randomized controlled trial on the effectiveness of Diclectin for the treatment of NVP had been done in order to support its reintroduction back into the US market until recently. In 2010, Koren et al. took this first step by evaluating the effectiveness of Diclectin as compared with placebo for NVP in a randomized, double-blind, multicenter placebo-controlled trial [54]. Women received Diclectin (*n* = 133) or placebo (*n* = 128) for 14 days, with symptoms of NVP evaluated using the 2-part PUQE score (clinical and quality of life). The subjects were instructed to take two tablets of Diclectin at bedtime on Day 1. If symptoms persisted into the afternoon of Day 2, the subject was instructed to take two tablets at bedtime on Day 2 followed by an additional tablet in the morning of Day 3. If assessment of the subject on Day 4 warranted an additional tablet to control evening symptoms, a fourth tablet was added in the mid-afternoon. The minimum dosage was 2 tablets at bedtime and the maximum was 4 tablets a day. Results showed that women receiving Diclectin had a significant greater improvement in symptoms when compared to placebo when considering the PUQE score and assessment of quality of life from day 1 to 15, as well as in day-to-day improvement in symptoms and well-being. In addition, approximately 48.9% of women opted to continue compassionate use of Diclectin in comparison to 32.8% of placebos, and women receiving placebo were 50% more likely to report use of alternative therapies to relieve symptoms when compared to the Diclectin group [54]. Finally, Diclectin was not associated with an increased risk of any adverse effects when compared to placebo. They concluded that Diclectin delayed-release formulation was both effective and well tolerated in the treatment of NVP.

After initiating treatment of NVP with a combination of doxylamine and pyridoxine, breakthrough nausea and vomiting can be treated with the addition of a different antihistamine or a dopamine antagonist. Dimenhydrinate is an H1-receptor antagonist that is widely used for the treatment of NVP. In addition, it is often useful to initiate treatment with an H2-receptor antagonist as their safety and efficacy are evident when using these agents for treating the reflux and heartburn symptoms associated with NVP [61]. Cimetidine, ranitidine, or famotidine are H2-receptor blockers that can be used especially if the patient has a history GERD, gastroduodenal ulcers, or other GI disease. Another second-line choice of therapy for NVP and HG in addition to dimenhydrinate is promethazine. Promethazine belongs to a class of dopamine (D2) receptor antagonists called phenothiazines that exhibit antiemetic properties by inhibiting gastric motility through the D2 receptors located in the GI tract and by inhibiting the chemoreceptor trigger zone [23, 49, 50]. Numerous human and animal studies, including those in the first trimester, show a lack of association between the use of promethazine during pregnancy and an increased risk for malformations [50]. Metoclopramide is another dopamine receptor antagonist that works as both an antiemetic and prokinetic by decreasing gastrointestinal emptying time and acting on the central chemoreceptor trigger zone [15, 23, 50]. Metoclopramide is particularly useful in patients where gastric dysrhythmia and gastric stasis are factor, that is, diabetic patients [13]. Both promethazine and metoclopramide can be added in the presence or absence of dehydration when antihistamines fail to treat the nausea and vomiting. Although data is limited, no animal or human studies have shown an increased risk for birth defects in animals and humans with these dopamine receptor antagonists. Matok et al. investigated the safety of metoclopramide during the first trimester in a retrospective cohort study of 3458 infants exposed to metoclopramide [62]. They found that exposure to metoclopramide in the first trimester was not associated with a significantly increased risk of adverse outcomes, including major congenital malformations, low birth weight, preterm delivery, and perinatal death.

Serotonin 5-hydroxytryptamine3-receptor (5-HT3) antagonists have been primarily used for the treatment of chemotherapy-induced nausea and vomiting. However, the use of ondansetron for NVP and HG is widely accepted. Although ACOG Guidelines recommend the use of serotonin 5-HT3 antagonists as a third-line pharmacotherapeutic intervention for NVP and HG, ondansetron is commonly used earlier in treatment due to less sedating effects when compared to promethazine [4]. Ondansetron works both centrally and peripherally at the 5-HT3 receptors located in the small bowel, vagus nerve, and at the chemoreceptor trigger zone, resulting in decreased stimulation of the medullary vomiting center [49]. Despite a lack of evidence on its use in pregnancy, data to date have been favorable. There has been no evidence of teratogenicity in animal studies even at doses significantly higher than that used in humans or in case reports of use in the first trimester [63–65]. In a prospective comparative observational study involving 169 infants exposed to ondansetron in the first trimester, 3.6% had major malformations, which was not significantly different from the rates in 2 control groups [65].

## 6. Conclusion

Although NVP and HG are two of the most common medical conditions of pregnancy, management can be very challenging for the clinician. Not only is appropriate diagnosis essential in order to initiate treatment, but timing of diagnosis is just as crucial to avoid delay in management. There are multiple pharmacotherapies available today, and each treatment regimen should be tapered to the particular patient. Due to the favorable effectiveness and safety profiles of the over-the-counter combination of pyridoxine and doxylamine in the USA and Diclectin in Canada, initiation with these medications early on is reasonable and recommended.

## Figures and Tables

**Table 1:** Differential diagnosis of NVP.

Peptic ulcer	Urinary tract infection
Hepatitis	CNS abnormality
Pyelonephritis	Preeclampsia
Pancreatitis	Acute fatty liver of pregnancy
Cholecystitis	Gastroesophageal reflux disease
Appendicitis	Mallory-Weiss tear
Gastroenteritis	Hyperthyroidism
H. pylori infection	

**Table 2:** Early pharmacotherapies for NVP/HG.

*First-line therapy:*
Pyridoxine hydrochloride monotherapy 10–25 mg po tid-qid
-or-Pyridoxine hydrochloride 10–25 mg po tid or qid *plus* Doxylamine succinate 25 mg 1/2 tablet po tid-qid
-or-Diclectin (pyridoxine hydrochloride 10 mg *plus* doxylamine succinate 10 mg) 4 tablets qd given 1 qam, 1 at lunch, and 2 qhs with a maximum of 8–12 tablets qd if increased BMI

*Breakthrough therapy:*
Dimenhydrinate 50–100 mg po/iv q 4–6 hours
Promethazine 12.5–25 mg po/iv/pr q 4–6 hours
Metoclopramide 5–10 mg po/iv tid
Ondansetron 4–8 mg iv/po tid
